# Avocado–Soybean Unsaponifiables: A Panoply of Potentialities to Be Exploited

**DOI:** 10.3390/biom10010130

**Published:** 2020-01-13

**Authors:** Bahare Salehi, Antonio Rescigno, Tinuccia Dettori, Daniela Calina, Anca Oana Docea, Laxman Singh, Fatma Cebeci, Beraat Özçelik, Mohammed Bhia, Amirreza Dowlati Beirami, Javad Sharifi-Rad, Farukh Sharopov, William C. Cho, Natália Martins

**Affiliations:** 1 Student Research Committee, School of Medicine, Bam University of Medical Sciences, Bam 44340847, Iran; bahar.salehi007@gmail.com; 2 Department of Biomedical Sciences, University of Cagliari, 09042 Monserrato (CA), Italy; rescigno@unica.it (A.R.); dettorit@unica.it (T.D.); 3 Department of Clinical Pharmacy, University of Medicine and Pharmacy of Craiova, 200349 Craiova, Romania; calinadaniela@gmail.com; 4 Department of Toxicology, University of Medicine and Pharmacy of Craiova, 200349 Craiova, Romania; daoana00@gmail.com; 5 G.B. Pant National Institute of Himalayan Environment & Sustainable Development Kosi-Katarmal, Almora, Uttarakhand 263643, India; laxmansingh@kunainital.ac.in; 6 Department of Nutrition and Dietetics, Bayburt University, 69000 Bayburt, Turkey; fatma.cebeci@gmail.com; 7 Department of Food Engineering, Faculty of Chemical and Metallurgical Engineering, Istanbul Technical University, Maslak, 34469 Istanbul, Turkey; ozcelik@itu.edu.tr; 8 Bioactive Research & Innovation Food Manufac. Indust. Trade Ltd., Katar Street, Teknokent ARI-3, B110, Sarıyer, 34467 Istanbul, Turkey; 9 Universal Scientific Education and Research Network (USERN), 1634764651 Tehran, Iran; mohamadbahia1996@gmail.com; 10 Department of Medicinal Chemistry, School of Pharmacy, Shahid Beheshti University of Medical Sciences, 11369 Tehran, Iran; amirrezadowlati@gmail.com; 11 Phytochemistry Research Center, Shahid Beheshti University of Medical Sciences, 1991953381 Tehran, Iran; 12 Department of Pharmaceutical Technology, Avicenna Tajik State Medical University, Rudaki 139, 734003 Dushanbe, Tajikistan; 13 Department of Clinical Oncology, Queen Elizabeth Hospital, 30 Gascoigne Road, Hong Kong 999077, China; 14 Faculty of Medicine, University of Porto, Alameda Prof. Hernâni Monteiro, 4200-319 Porto, Portugal; 15 Institute for Research and Innovation in Health (i3S), University of Porto, 4200-135 Porto, Portugal

**Keywords:** avocado–soybean unsaponifiables, tocopherol, phytosterols, carothenes, osteoarthritis, scleroderma, menopause

## Abstract

Avocado and soybean unsaponifiables (ASU) constitute vegetable extracts made from fruits and seeds of avocado and soybean oil. Characterized by its potent anti-inflammatory effects, this ASU mixture is recommended to act as an adjuvant treatment for osteoarthritic pain and slow-acting symptomatic treatment of hip and knee osteoarthritis; autoimmune diseases; diffuse scleroderma and scleroderma-like states (e.g., morphea, sclerodactyly, scleroderma in bands). Besides, it was reported that it can improve the mood and quality of life of postmenopausal women in reducing menopause-related symptoms. This article aims to summarize the studies on biological effects of the avocado–soybean unsaponifiable, its chemical composition, pharmacotherapy as well as applications in autoimmune, osteoarticular and menopausal disorders. Finally, we will also discuss on its safety, toxicological and regulatory practices.

## 1. Introduction

Avocado and soybean unsaponifiables (ASU) constitute vegetable extracts made from fruits and seeds of avocado and soybean oil. It is mainly prepared in the ratio of 1:2, though many other ratios have been tested (i.e., 1:1; 1:3; 2:1) [1,2,3]. This ASU mixture was first manufactured in France and commercialized as Piascledine^®^300 (Laboratories Expanscience, Courbevoie, France) [1]. ASU is a complex mixture of avocado oil, containing polyols (15%), sterols (4% to 20%), long-chain saturated hydrocarbons (5%), squalene (2%), and tocopherols (in trace amounts). On the other hand, the key constituents in soybean oil contains mainly sterols (40% to 50%), tocopherols (10%), terpene alcohols (1% to 10%), methylsterols (<5%), squalene (4%), saturated hydrocarbons (1%), and aliphatic alcohols (<1%) [4]. However, until now the identity of the active constituents of the ASU extract still remains unclear [5]. In fact, some reports have suggested that the phytosterols (i.e., β-sitosterol, campesterol, and stigmasterol) and isoflavones (i.e., daidzein, genistin, and glycetin) present in ASU extract play a key role in preventing the development of osteoarthritis, rheumatoid arthritis, osteoporosis-related fractures in postmenopausal women, cardiovascular diseases, hypercholesterolemia and atherosclerosis [6].

Many food supplements based on the unsaponifiable fraction of avocados and soybeans (ASU) can be found in the market. Some formulations contain only ASU extract, while others mixed with extracts from other plants (e.g., *Uncaria tormentosa* and *Zingiber officinalis*) [7].

ASU is formulated into capsules to restore the normal cartilage structure by stimulating the proteoglycans and collagen synthesis [8]. In addition, the therapeutic indications for ASU, as well as packaging and dosage, vary from country to country. For instance, in France, the Haute Autorité de Santé (HAS) recommends ASU therapy in rheumatology as adjuvant treatment for osteoarthritis pain and as symptomatic slow-acting treatment for hip and knee osteoarthritis [9]. In Italy, the Italian Drug Agency (Agenzia Italiana del Farmaco, AIFA) suggests ASU as an adjuvant therapy for diffuse scleroderma (cutaneous and esophageal manifestations), scleroderma-like states such as morphea, sclerodactyly, scleroderma in bands, post-phlebitic and post-varicose status, and for hemorrhagic and painful phenomena of periodontal disease [9].

The biological properties of ASU action can be characterized by—an increase in the collagen content in tissues, an increase in tissue lipids, and a significant increase in the proportion of extractable constituents in relation to insoluble ones, with a considerable increase in both tissue proteases and collagenases activity of serum leucine-peptidase [8,10]. With regards to unsaponifiable extracts, they contain substances characteristic of soybean and avocado seed extracts, which modify the metabolism of connective tissue. Avocado extract stimulates stroma-related enzymes, while soybean extract alone sensitively stimulates lysosomal enzymes with an acidic pH and, to a lesser extent, some neutral lysosomal proteases [11]. Consequently, the association of both extracts which constitute ASU exerts more powerful synergistic effects, different from that exercised by each of the individual components [12]. The action of both unsaponifiables has also been confirmed in granulomas composition, The increase in the ratio of the macromolecules (collagen, glycoproteins) in both the soluble and insoluble fractions of granuloma extracts can be interpreted as a sign of the increased degradation of these tissue constituents [13]. The favorable effects observed after ASU administration and can be attributed to a collagenolytic effect [9].

Regarding little information on the vast health beneficial properties of ASU extract, the current review aims to provide updated information on the health benefits of ASU extract, not only in osteoarthritic disorders and autoimmune diseases but also cover other disorders.

## 2. Avocado–Soybean Unsaponifiables: Extraction, Analysis and Chemical Compounds

### 2.1. Extraction and Analysis of ASU

Edible oils and fats have been used for a long time. From the lipid soap production, it was clear that some lipids escaped from the chemical saponification process. According to this observation, natural oils and fats are characterized by the unsaponifiable fraction [14]. In fact, lipids present in animal or vegetable have a remarkable variety of physical forms, and can be present as aggregates, or they can be associated with proteins and carbohydrates, e.g., the biological membranes.

#### 2.1.1. Extraction Methods of ASU

ASU is prepared in two steps: (i) obtain the avocado and soybean crude oils by cold pressing through mechanical procedures at temperatures below 50 °C; (ii) extraction of unsaponifiable lipids: molecular distillation of crude avocado and soybean oil, saponification, extraction, purification.The extraction methods normally used when lipids subjected to further characterization of the fat which called cold methods.Since the extraction conditions of these methods limit the oxidative processes of the lipidic fractionas much as possible, allowing one to preserve the original composition [15]. These methods are generally based on the use of a binary mixture of solvents, such as chloroform/methanol [16,17], dichloromethane/methanol, or hexane/isopropanol [18].

Molecular distillation is a method of separating the unsaponifiable substances from the crude avocado oilat low pressures. It is based on the intensification of the four elementary processes—the diffusion of components through liquid, the vaporization ofthe surface of the liquid, vapor transport to the condensation surface, and condensation on the surface of the capacitor. Separation by molecular distillation is useful for the purification and concentration of unsaponifiable, low-vapor pressure thermoset substances. This method is applied when the conventional distillation methods lead to the thermal degradation of the products or if the vapor pressure of the components separately is verylow that separation at atmospheric pressure or at medium vacuum would require extremely high temperatures.

Consequently, at the end of the saponification process of vegetable oils by extraction with an organic solvent, an unsaponifiable fraction could beobtained. 

#### 2.1.2. Analysis Methods of ASU

The extracted lipids then can be further fractionated by means of chromatographic techniques, such as thin layer chromatography (TLC), gas–liquid chromatography (GLC), and high-performance liquid chromatography (HPLC). In reality, chromatographic techniques can be combined with sophisticated analytical techniques, such as mass spectrometry (MS). In addition, nuclear magnetic resonance (NMR) is becoming routine for the study of the lipid fraction in biological matrices.

### 2.2. Chemical Composition of Avocado–Soybean Unsaponifiables

Typically, from this extraction process, a complex mixture of compounds is obtained, the main classes are tocopherols and tocotrienols, phytosterols, carotenes, chlorophylls and a mixture of other unsaponifiable compounds [15].

#### 2.2.1. Tocopherols and Tocotrienols

Tocopherols and tocotrienols have α-, β-, γ-, and δ-isomers that differ in number and position of the methyl groups in the chromane ring and constitute a series of benzopyranols that present in plants and photosynthetic organisms. The synthesis starts from homogentisic acid with a complex series of reactions [19]. Taken together, these two groups of molecules are called tocochromanols. Tocotrienols have an unsaturated farnesyl isoprenoid tail with three *trans* double bonds, whereas tocopherols have a saturated phytyl tail (Table 1). Tocochromanols are often found in chloroplasts and, collectively, they have been termed vitamin E (the individual tocopherols are properly called ‘vitamers’) though only α-tocopherol has this designation, because of its biological activity and presence in the human body [20].

The properties of vitamin E have been known for many decades. Many studies have analyzing the effects of vitamin E in cardiovascular diseases [21], immunity [22], treating and preventing osteoarthritis [23]. However, interest in tocotrienols has awakened due to their biological effects and the therapeutic properties [24,25]. Many studies have shown that tocotrienols are useful in the treatment of high cholesterol levels due to their ability to inhibit the key enzyme of cholesterol biosynthesis, HMG-CoA reductase [26]. The tocotrienols also show excellent antioxidant properties thanks to their lateral unsaturated tail, which allows easier access to the lipid bilayer of biological membranes [27]. Moreover, the anti-cancer [28] and neuroprotective [25] properties of tocotrienols have also been documented.

#### 2.2.2. Phytosterols

**Figure 1:**
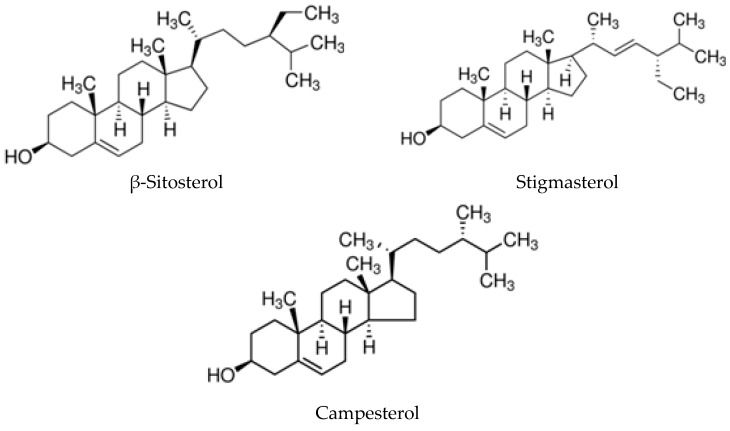
Chemical structure of the three most representative phytosterols in vegetable oils.

Phytosterols are bioactive sterols present in vegetables, especially in natural oils, nuts and cereals, and are structurally similar to sterols from animal sources. Compared to cholesterol, they have an additional methyl or ethyl group in their side chain. The absorption of dietary plant sterols in humans is low compared to cholesterol [29]. All phytosterols, some of which are depicted in Figure 1, have a hydroxyl group at the 3-position. In oils, the sterol hydroxyl group is not linked to any other moiety, but phytosterols are usually present as conjugates with the hydroxyl group covalently bound via an ester bond to a fatty acid. When the double bond in the sterols is saturated, the resulting compounds are termed stanols [30].

Phytosterols are amphiphilic and important constituents of all membranes, especially the plasma membrane, mitochondrial outer membrane and endoplasmic reticulum. They can regulate membrane fluidity and permeability in plasma membranes by restricting the mobility of fatty acyl chains in a similar manner to cholesterol in mammalian cells [31]. Phytosterols present in our diet are well-known for their inhibitory effects on intestinal cholesterol absorption and in decreased LDL-cholesterol levels [32]. Furthermore, evidence is accumulating that these compounds have effects beyond cholesterol-lowering effects. Recently, Plat et al. (2019) reviewed the possible side effects in the field of immunology, hepatology, gastroenterology and rheumatology arising from the increasing consumption of foods rich in plant sterols and stanols [33]. The emerging scenario is that, along with multiple positive health effects, an excessive intake of phytosterols and phytostanols requires further investigation to understand the complete health effects of plant sterols and stanols in both healthy individuals as well as in individuals suffering from specific diseases.

#### 2.2.3. Carotenes, Chlorophylls, and Other Unsaponifiable Compounds

The main pigments found in vegetable oils are carotenoids and chlorophylls [34]. Carotenoids cover a wide range of functions in human health [35]. They primarily exert antioxidant effects, but individual carotenoids (α- and β-carotene, lutein, zeaxanthin, lycopene) may also act through other mechanisms as in eye function [36]. There is evidence that carotenoids could improve cognitive function, skin-UV protection, and even may prevent some types of cancer [37,38].

Chlorophyll is an ester of chlorophyllic acid and phytol alcohol [39]. It occurs in several distinct forms; chlorophylls *a* and *b* are the major types typical of higher plants and green algae; chlorophylls *c* and *d* are found in different algae. Bacterio-chlorophyll occurs in certain bacteria [40]. The chlorophyll molecule consists of a central magnesium ion surrounded by a nitrogen-containing structure, a porphyrin ring; attached to the ring is the phytol chain. The variations are due to minor modifications of certain side groups [41].

It has long been debated whether chlorophylls, and their natural or synthetic derivatives, could be absorbable by humans, despite the fact that their consumption has been documented for a long time in traditional medicine [42,43,44]. However, recent research has confirmed that chlorophyll derivatives are absorbable by the human intestine [45]. The biological activities attributed to chlorophylls are various. Particularly interesting are those consistent with cancer prevention, such as antioxidant and antimutagenic effects, mutagen trapping, modulation of xenobiotic metabolism, and induction of apoptosis [46].

A miscellany of other compounds, extractable in organic solvents, can be found in the unsaponifiable fraction of numerous plants and their composition varies according to the plant being considered. It is necessary to consider that among these compounds, present in vegetable oils, hydrocarbons may also be present since they do not saponify. For example, squalene is present in some vegetable oils, where it can also represent 50% of the unsaponifiable fraction, such as in olive oil.

#### 2.2.4. Main Components of ASU

An ASU fraction is obtained by the purification and fractionation of the respective oils [47]. The quantity and composition of extractable lipids depend on various factors, including the botanic cultivar, harvesting time, and the growth condition of the plant.

At 20 °C, ASU looks like an oily and thick paste, insoluble in water. Studies performed on these vegetable oils showed that unsaponifiables differ in the content of total unsaponifiables and, also, in their relative abundance [48]. Avocado oils contain more unsaponifiables than soybean oils (4.8%–12.2 % from the fruit flesh oils), whereas unsaponifiables account for over 50% of the oils from the avocado kernel. Total sterols were also more represented in avocados (3770–10720 μg/g oil) than in soybeans (on average 3600 μg/g oil). Concerning the sterol composition of avocado oils, β-sitosterol was the dominant one (ca. 90 %), with a limited amount of campesterol and stigmasterol. The sterol fraction of soybean oil had about 50% β-sitosterol and the remaining 50% was almost equally divided between campesterol and stigmasterol [49]. The tocopherol content was at least ten times higher in soybean oils (1130–1450 μg/g oil) than the content of both avocado flesh and kernel oil. Soybean tocopherols were rich in γ-tocopherol (>66 %), whereas δ-tocopherol was >21% and α-tocopherol was 11%. Interestingly, α-tocopherol accounted for 100% of the total tocopherols in avocado oils [50,51].

Dijkstra (2016) reported the absence of tocotrienols in soybean oil [46]. Tocopherols were found in soybean oil, by means of a simultaneous analytical method HPLC-DAD-FLD based, and also confirmed the absence of tocotrienols [52]. The unsaponifiable fraction of avocado contains tocotrienols, but in very low amounts. A study aimed at determining the tocochromanol content in raw and processed fruits and vegetables revealed the presence of tocotrienols in quantities of about 1% of the total tocochromanols, only in some avocado cultivars [53]. Tocotrienols have been shown to possess higher antioxidant and anti-inflammatory effects than α-tocopherol [24].

The presence of total sterols can also vary depending on the different growth conditions and the variety considered. De Souza et al. (2015) extracted by centrifugation the most cultivated avocado varieties in Brazilian territories and compared them to a commercial product. The Margarida and Hass varieties showed a phytosterol content that reached almost 100 mg/100 mL of oil [54]. Such a high amount of phytosterols makes ASU an important vehicle for bioactive natural compounds for human health.

As previously stated, the content of the various lipid classes of the unsaponifiable fraction mainly depends on plant variety, and on the purification and fractionation process adopted. However, although the ASU is chemically well characterized, it is not possible to exclude the fact that a part of its therapeutic action may be mediated by unidentified factors. Therefore, it is preferable to use the unsaponifiable fraction “in toto”, which, in this way, can preserve the glycosylated fraction [55].

## 3. Avocado–Soybean Unsaponifiables for Medical Purposes

### 3.1. Osteoarticular Disorders

#### 3.1.1. A Brief Overwiev Of Ostheoarticular Disorders

Osteoartrithis (OA) is a form of arthritis that causes cartilage degeneration, especially in the elderly [56]. This chronic joint disease has as the main symptoms pain and stiffness due to the erosion of articular cartilage, bone remodeling, new bone formation, and synovial inflammation [57]. Rheumatoid arthritis is also associated with the time-span joint degradation, with symptoms such as articular cartilage erosion, synovial inflammation, and subchondral bone alterations, resulting in severe pain and impaired joint function [58].

In normal conditions, from the molecular point of view, the equilibrium between extracellular matrix components (ECM) is due to the synthesis and degradation of collagen type II and aggrecan from articular chondrocytes [59]. When chondrocyte metabolism is unbalanced, an abundant production of inflammatory cytokines and matrix-degrading enzymes occur, simultaneously to a down-regulation of anabolic signaling, leading to the destruction of the ECM [60,61]. Inflammatory cytokines, chemokines, and other inflammatory mediators are produced from chondrocytes, but also from synovial cells and other joint tissues [62].

In chronic osteoarticular disorders, there is an abnormally regulated function of cytokines and growth factors, prostaglandins, cartilage matrix fragments, reactive oxygen intermediates, proteolytic enzymes and protease inhibitors [63,64] by a series of biochemical events, including the abundant production of proinflammatory cytokines IL-1b, IL-6 and tumor necrosis factor-alpha (TNF-a). The overproduction of these cytokines inhibits the production of proteoglycans and type II collagen and the proliferation of chondrocytes, while upregulation leads to formation of matrix-degrading enzymes, such as matrix metalloproteinases (MMPs) that, in turn, degrade cartilage [65]. These inflammatory mediators induce a downstream signaling pathway, which is abnormally activate in the NF-KB and MAPK pathways in osteoarthritis chondrocytes to express MMP, NOS2, COX2 and IL-1 [66].

The drugs that belong to the group of structurally modifying drugs represent, theoretically, the ideal osteoarthritis medication, as they are the only medication capable of preventing joint damage and restoring cartilage structure. These are represented by glucosamine sulfate, chondroitin sulfate and ASU. The mode of action of the drugs that can modify the structure of the articular cartilage in OA is realized on several planes: it stimulates the synthesis (anabolic effect) of the chondrocytes of the extracellular matrix components and reduces their degradation (anti-catabolic effects) [67,68].

OA joint pain is controlled using analgesic drugs (acetaminophen), non-steroidal anti-inflammatory drugs (NSAIDs) (ibuprofen, naproxen) and topical analgesics. [69]. In the case of osteoarthritis, NSAIDS, such as aspirin, ibuprofen, diclofenac, celecoxib and naproxen have been prescribed to such patients to get rid of the pain associated with this disease [70,71]. However, it is not clear which one of these is better over another, but it is proven that the prolonged use of these drugs is known to have potent therapeutics fallouts, e.g., increases the risk of cardiovascular complications, kidney damage, and gastrointestinal problems [72,73]. Another class of analgesics used includes opioids that have been found to show great effectiveness, but they have also been reported to show cardiorespiratory complications and are known to develop tolerance [74]. This limited effectiveness of the above-mentioned drugs prescribed and the associated adverse effects has necessitated the deepening of the understanding regarding safer natural analgesic therapies. These therapies are aimed to develop novel and more effective causative agents, with limited side effects.

#### 3.1.2. *In Vitro and In Vivo* Studies of ASU in Osteoarticular Disorders

**Figure 2:**
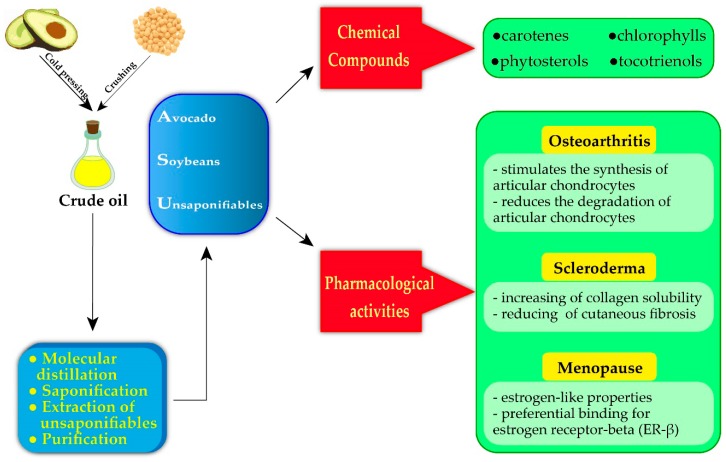
Extraction of ASU, chemical composition and the main pharmacological activities for human health.

ASU has been used in numerous experimental studies to test its possible biological effects. A recent experimental evidence has recommended the use of ASU extract (herbal remedies) as a potent therapeutic agent for various arthritic diseases [1]. So, ASU has been studied for its anti-inflammatory, anti-catabolic, and anabolic effects on cartilage metabolism, principally on chondrocytes [68,75]. Some studies have explored the action of ASU that seems to act on different molecular mediators implicated in various target tissues/organs (Table 2, Figure 2).

The most important results of these experimental studies highlight the reduction of pro-inflammatory cytokine mediators: interleukins IL-1β, IL-3, IL-6, IL-8, IL-13, prostaglandin 2 (PGE 2), transforming growth factor-β (TGF-β) with its isoforms TGF-β1 and TGF-β2 [76,77], proteolytic enzymes (e.g., MMPs) [87], and the mediators involved in the synthesis of various reactive oxygen species (ROS, e.g., inducible nitric oxide synthase) [58,78,79].

The molecular mechanism of ASU involves the inhibition of NF-κB activation. NF-κB is a transcription factor that regulates the inflammatory response in chondrocytes. It normally resides in the cytoplasm, but, once activated, it moves towards the nucleus to induce the expression of pro-inflammatory genes, including enzymes that degrade the cartilage matrix [75]. Likewise, ASU reversed the catabolic effect of IL-1b in human fibroblasts by inducing a significant decrease in MMP-2, MMP-3, and tissue inhibitors of matrix metalloproteinase-1 in the presence of IL-1b [80]. The mechanism of action of ASU in OA is not well elucidated, but there is some evidence regarding its ability to inhibit MMPs and stimulate the synthesis of TGF-β, which plays an important role in cartilaginous tissue homeostasis. ASU has inhibitory effects on inflammatory and catabolic mediators, thus preventing cartilage degradation. It inhibits the expression and production of cytokines, chemokines, PGE2, nitric oxide, and MMPs. In human articular chondrocytes stimulated in cultures with IL-1b, ASU suppressed IL-6, IL-8, MIP-1β, PGE2, and NO production (Table 1) [67,68].

For instance, some in vitro studies reported that ASU extract is capable of stimulating matrix production and reducing the deleterious effect of IL-1, possibly by producing TGF-β. ASU is also known to stimulate and restore the aggrecan production, even after IL-1β treatment, decrease the matrix metalloproteinase-3 (MMP-3) production and stimulate the tissue inhibitor of metalloproteinases- 1 (TIMP-1) production.

ASU in different ratios were studied on metalloproteinase activity, cytokines and prostaglandins levels by chrondrocytes. A study showed that different ratios of ASU mixtures provided a reduction in stromelysin production, interleukins IL-6, IL-8 and prostaglandin PGE2 by chrondrocytes. The study reported that ASU could partially reverse the IL-1 effects in chondrocytes [67]. Another study also reported a modulating effect of ASU on VEGF and TIMP-1 and chemo invasion and suggested that ASU might have a role in the treatment of rheumatoid arthritis by controlling invasiveness [81].

Ownby et al. (2014) made a mixture from ASU and epigallocatechin gallate (EGCG) extract and studied the responsiveness of articular chondrocytes of the carpal joints of mature horses and tested its ability to inhibit joint inflammation [78]. The results highlight marked inhibitory effects on cytokine gene expression (i.e., IL-1β, IL-6, IL-8), TNF-α, PGE-2 and cyclooxygenase-2 (COX-2) through the modulation of NF-κB.

Likewise, Grzanna et al. (2010), using monocyte/macrophage cell models, determined the inhibitory effects of ASU extract (8.3 µg/mL) in combination with chondroitin sulfate (CS) (20 µg/mL) on both the synthesis and expression of pro-inflammatory stimulators and mediators, such as cytokine expression and PGE-2 production [88]. An addition, Grzanna et al. (2018) carried out another study where ASU extract was used with glucosamine (GLU), CS and with or without the presence of carprofen, a nonsteroidal anti-inflammatory drug (NSAID) [89]. The aim of this study was to assess the anti-inflammatory activity using a composite mixture of ASU extract (8.3 μg/mL), GLU (11 μg/mL), CS (20 μg/mL) and/or the NSAID carprofen (40 ng/mL). Briefly, it was stated that when only ASU+GLU+CS was used, the resultant activity was less significantly enhanced than when used with carprofen. Thus, as the main findings of this study show, the use of low doses of NSAIDs with the above-mentioned concentrations of ASU+GLU+CS is recommended, since it seemed to produce much better, safe and effective treatments for joint pain. These data suggest that ASU may have structure-modifying effects in OA by inhibiting cartilage degradation and promoting cartilage repair [67].

A recent study on a guinea pig model investigated the therapeutic effects of an extract of guava leaf on experimentally induced OA, and also used ASU. The study induced OA in 30 male guinea pigs and followed the pigs for 8 weeks until OA was confirmed by radiography and histopathology. It was found that ASU reduced the OA severity when compared to the control group [90]. Hashemibeni et al. (2018) compared the efficacy of ASU and TGFβ1 on chondrogenic differentiation of human adipose-derived stem cells. The study reported that ASU improved proliferation and increased the survival of differentiating chondrocytes in fibrin scaffolds more effectively than TGFβ1 [10]. The findings suggest that ASU acts on multiple cell types implicated in inflammation; on the other hand, ASU has beneficial effects in different sites on joints involved principally in OA. At a clinical level, ASU shows a great ability to reduce pain and stiffness. It is not clear which components present in ASU is responsible for these effects. Studies have shown the anti-inflammatory, antioxidant and analgesic properties of phytosterols [91]. Further studies are needed to verify the mechanisms and the specific molecules implicated at the cellular and metabolic level.

#### 3.1.3. Clinical Efficacy of ASU in Osteoarthritis

Some clinical studies were focused on the effect of ASU on NSAID or analgesic use in OA patients, but some investigated the structural effects of ASU in the treatment of patients with symptomatic OA.

Blotman et. al. (1997) studied 163 patients with primary femoro-tibial or hip osteoarthritis in a randomized double-blind study placebo-controlled study. The patients received either one capsule of ASU or a placebo for 3 months. The study showed that ASU reduced the need for NSAID for lower limb osteoarthritis patients [71].

A double-blind study conducted on 260 patients with femoro-tibial knee OA and on NSAIDs and/or analgesics and compared the symptomatic effects of 300 or 600 mg daily of ASU to placebo. The results show that the daily intake of 300 or 600 mg of ASU for 3 months has significant effects compared to the placebo. NSAID and analgesic intake by patients showed a 50% and 71% decrease with 300 and 600 mg ASU intake, respectively, compared to a 36% decrease with placebo. The study concluded that daily ASU intake was more effective than placebo and that ASU dosage did not matter [92].

Lequesne et al. (2002) also investigated the structural effect of ASU on hip OAin 108 patients (ASU group = 55, placebo group = 53). The overall results show that there was no significant difference in joint space width (JSW) between the ASU and placebo groups. However, the study reported that the joint space loss was significantly higher in placebo than in ASU group, when only the most seriously affected subgroups were evaluated [93].

Pavelka et al. (2010) compared the effect of 300 mg ASU (once daily) with 400 mg CS (3 times daily) for femoro-tibial OA patients in a 6-month study. In this study, 183 individuals taking ASU and 178 taking CS were screened. To assess the effect, the authors used the Western Ontarion and McMaster Universities Osteoarthritis (WOMAC) index primarily followed by the Lequesne index, but there were no detected differences between ASU administration or CS intake in terms of efficacy and safety [2].

Another study included 60 patients with knee OA. The patients were given either ASU (300 mg daily) or diclofenac (25 mg, 3 times/day) for 8 weeks and results were estimated using WOMAC index. The study suggested that ASU can be a promising substitute to NSAIDs, due to its better patient compliance and WOMAC score [94]. Another study observed 345 patients with symptomatic hip OA in a 3-year randomized double-blind placebo-controlled study. Patients were screened for standing pelvis, target hip anteroposterior (AP), and oblique views were taken every year. The study could not find a significant difference in the mean JSW loss between the ASU and placebo groups but reported 20% fewer progressors in the ASU than in the placebo groups (40% vs 50%, respectively, *p* = 0.040) [1].

### 3.2. Autoimmune Disorders

The human immune system is known to protect the body against the foreign invaders that basically exert a wide variety of deleterious effects [95]. Sometimes the optimal functioning mechanism of the immune system is said to act as a ‘double-edged sword’, either by healing the physiological state or by damaging it. The act of immune dysfunction acting against its own normal components of the body results in autoimmune disorders [96]. Many pieces of conclusive experimental evidence have suggested that they result from the interaction between various genetic and environmental factors, and even the distinct functioning of the endocrine system [97].

The known etiology of these autoimmune dysfunctions is associated with the overproduction and up-regulation of several pro-inflammatory substrates, such as interleukin 1β (IL-1β), a stimulant cytokine which further stimulates the synthesis of other pro-inflammatory cytokines, such as interleukin-6 (IL-6), interleukin-8 (IL-8), macrophage inflammatory protein (MIP), and reactive oxygen species (ROS), such as NO^.^, O_2_^−^, H_2_O_2_ [98]. Other components that lead to this immune disorder include tumor necrosis factor-α (TNF-α) and -β (TNF-β), prostaglandin E-2 (PGE-2), inducible nitric oxide synthase (iNOS), metalloproteinases, including collagenases (MMP-1, 8, 13), aggrecanases (ADAM-TS4), stromelysin-1 (MMP-3), and gelatinases (MMP-2) [68,80].

Scleroderma is a rare autoimmune disorder, in which skin and connective tissue gets thickened due to too much collagen production. In scleroderma, the collagen content of the skin increases while the number of adipocytes decreases.

ASU was suggested by Jablonska et al. as being an effective agent in the treatment of scleroderma by increasing the collagen solubility and reducing cutaneous fibrosis. A larger cohort study is needed to investigate the effect of ASU in the treatment of scleroderma [82].

The ASU effect on autoimmune disorders seems to be an unexplored subject. However, considering the soybean content of ASU, it is worth mentioning the effects of soy on many immune disorders. A crossover randomized clinical trial was performed with 14 diabetic patients and reported that soy protein inclusion in the diet was beneficiary to the serum lipid profile and renal function. This effect was attributed to the isoflavones in soy protein [99]. Another study conducted on eight people reported that consuming soy protein as half of the daily protein intake did not show any significant effects on renal function or proteinuria. However, the study reported a significant association between soy protein intake and reduction in serum cholesterol and triacylglycerol concentrations [100]. It should be noted that there is some conflict regarding the effects of phytoestrogens of soy on immune disorders. A mouse model study on lupus disease reported that a soy diet compared to a casein diet worsened the clinical course of lupus [101].

ASU could also be useful to treat inflammatory bowel disease. The supplementation of soy isoflavones to neonates and piglets was also suggested to reduce the intestinal barrier damages of lipopolysaccharide [102]. A study using a pig model of intestinal inflammation tested the effect of soy-derived di- and tripeptides and reported the anti-inflammatory effects of these peptides in vivo [103].

### 3.3. Menopause

Menopause is described as the end of menstruating and is a normal condition that all women experience when they age [104]. In the initial days/years of menopause, the associated symptoms include hot flashes, vaginal dryness, and rapid bone loss as a result of osteoporosis and sleep disturbances [105]. Hormone replacement therapy (HRT) has been used as the most common therapy to get relief from menopausal dysfunction. But again, it comes with possible fallouts, such as breast or endometrial cancer, irregular bleeding, thromboembolic events, mastalgia, nausea, weight gain, migraine, among other issues. Presently, HRT is forbidden for women currently undergoing or who have a history of breast cancer, coronary heart disease (CHD), venous thromboembolic events or stroke, liver disease, mysterious vaginal bleeding, high-risk endometrial cancer, or transient ischemic attack [106].

In the light of these aspects, the use of other alternative therapies, possibly through the use of herbal formulations, is recommended. Recently, herbal remedies, particularly the ones inheriting phytoestrogens values, are in great demand for the treatment of such climacteric symptoms. Soy isoflavones and extracts are the preferred phytoestrogen sources, with estrogen-like properties. Phytoestrogens are the chief constituents of polyphenols, structurally similar to endogenous estrogen, but having weak estrogenic properties as compared to endogenous ones [105].Keeping this in mind, soybean rich in unique dietary phytoestrogens (i.e., isoflavones daidzein, genistin, and glycetin) has gained considerable importance; apart from this, gabapentin, clonidine, selective serotonin reuptake inhibitors (SSRI’s), black cohosh, and vitamin E are other alternatives used for conventional HRT [83]. These phytoestrogens are reported to have selective estrogen receptor modulators preferential for estrogen receptor-beta (ER-β) rather than for estrogen receptor-alpha (ER-α). As a result, when these phytoestrogens bound to ER-β trigger an effective transcriptional activity, either the response can be an agonist response or antagonist, depending on the compound (stimulus) and the site of action (target tissue) [84].

ASU is an herbal medicine derived from avocado and soy which is used to relieve hot flashes in menopausal women [85]. It is known that ASU has a potent phytoestrogenic value and exerts significant positive effects in reducing menopausal-related symptoms, such as hot flashes, besides being able to improve mood and quality of life in postmenopausal women [86].

However, there are conflicts regarding the effect of soybean on climacteric symptoms of menopause. Some authors have suggested positive effects [107], while others did not report any significant effect [108,109]. A study investigating the association between dietary fiber intake and serum estrogen levels also studied ASU intake. The study suggested that ASU is related to higher serum estrogen levels, but the source of the effect needs to be explored [110].

An open label randomized study included 49 women and tested the ability of ASU to relieve menopausal symptoms. The women were divided into two groups; one group received 1 mg ASU daily and another group were treated with HRT (0.625 mg conjugated estrogen and 2.5 mg medroxyprogesterone acetate tablets). The visual analog scale (VAS) was used to determine the intensity of hot flashes, and the climacteric symptom was determined by the Greene Climacteric Scale (GCS) and Blatt–Kupperman Menopausal Index (BKMI). No significant differences were stated in the hot flash severity decrease for the ASU and HRT groups (GCS; *p* = 0.571 and BMKI; *p* = 0.891) [85]. Thus, studies investigating the effect of ASU vs HRT are limited, but they report similar symptom relief effects. Concerning the HRT side effects, ASU seems a feasible alternative to HRT.

### 3.4. Other Pharmacotherapeutic Uses of ASU

There are studies that highlighted other beneficial effects of ASU on different medical situations, such as chronic dorsalgia [111], gingival inflammation, periodontitis [112] or back pain [113]. Other health benefits include its ability to decrease the risk of osteoporosis, heart disease, and breast cancer [114].

ASU was also studied for its effect in wound healing. A rat model study investigated the effect of ASU on rat wound healing by randomly dividing rats into three subgroups (20 rats each). Each rat in the control (saline), vehicle (cream) and treatment (cream plus ASU) groups were wounded on the dorsum (2×2 cm^2^ wound) and the wounds were screened daily. The study found that treatment (cream plus ASU) produced a significantly higher level of tissue glycosaminoglycan and collagen contents compared to controls. It was also reported that the treatment had a modulating effect on inflammation, improved fibroplasia and provided higher amounts of scar tissue; so, it was concluded that ASU is a promising agent in wound healing [115].

## 4. Avocado–Soybean Unsaponifiables: Safety, Toxicological and Regulatory Aspects

ASU is a natural therapeutic alternative recommended for arthritic pain [8]. The active ingredient of ASU-based products is a mixture of soybean and specific avocado unsaponifiables. [116]. More specifically, these are based on the avocado/soybean unsaponifiables ratio (1:2) and the specific composition of both unsaponifiables [116]. Natural remedies are popular in arthritis patients. Most people believe that natural products are safer than prescribed drugs. The explanation is that there are fewer undesirable side effects with natural products [117].

After oral administration, ASU can cause side effects, although they do not appear in all people. Lipid-odor regurgitation may rarely occur, which can be avoided by taking the capsule during the meal. Diarrhea and stomach pains may also occur. However, more severe side effects, such as lymphocytic colitis, have also been reported [118].

Hypersensitivity reactions may occur rarely [71]. Very rarely, plasma levels of liver enzymes (transaminases, alkaline phosphatase and gamma-glutamyl transpeptidase) may increase. Gluszko et al. (2016) demonstrated in a prospective observational study, which included 4822 patients diagnosed with knee OA, that ASU treatment was safe [118]. Adverse reactions occurred in a small number of patients: only five patients developed diarrhea, three had nausea, flatulence or abdominal pain and two had high blood pressure and headache [118].

Studies in laboratory animals have not revealed the potential teratogenic effects. At the moment, there is insufficient clinical data to evaluate eventual malformities or fetotoxic effects after ASU in pregnant women [119]. Therefore, it is not recommended to administer the medicines drug during pregnancy. Also in the absence of data on the excretion of the active substances in breast milk, it is not recommended to administer ASU during breast-feeding too [119].

Regarding safety, a few studies have reported that there were no significant differences in adverse effects between ASU and placebo. Two studies were conducted within 3 months for hip and knee OA, while the other study was carried out only for OA of the knee [1,8]. The results of both studies show that patients who took 300 mg of ASU daily did not need pain medications as much as before, meaning that the use of NSAIDs had decreased. No significant differences were observed between ASU doses ranging from 300 to 600 mg once daily [1,8].

In different countries (e.g., US and Europe), products containing ASU are classified differently, either as pharmaceutical agents or as nutritional supplements. In principle, different properties are usually ascribed to the two product classes. In the first case, the chemical composition should be very well defined, while, in the second case, the quality and composition of the marketed product may vary. Some nutritional supplements on the market contain additional active ingredients besides ASU. This makes the different formulations difficult to compare. It is now beyond doubt that ASU-based products exhibit significant positive biological effects in vitro. However, discrepancies in the results obtained have often been highlighted [9]. More studies are needed, especially in the long term and consolidate their use in humans.

## 5. Concluding Remarks and Perspectives

Over the years, the many health benefits of ASU extract have meant that it has gained a huge importance as a natural alternative therapy for various medical conditions, such as osteoarthritis and autoimmune disorders (rheumatoid arthritis, scleroderma, inflammatory bowel disease), and acts as a natural therapeutic alternative to hormonal replacement therapy in the case of menopausal women.

ASU has chondroprotective proprieties as well as anti-inflammatory effects. In order to develop more comprehensive and effective formulations, ASU extract along with other nutraceutical supplements were assessed to provide efficient alternatives to NSAIDS. Some of the recent studies in various arthritis cell lines (i.e., chondroblasts, fibroblast, osteoblasts and macrophages etc.), experimental animals (rats, dog, horse etc.) and human randomized clinical trials, have proven the beneficial effects of ASU alone and in combination with other substituents.

Considering its effect on OA and its role in providing positive structural changes, ASU seems to be a promising drug for OA.

Autoimmune dysfunction is associated with an overproduction and up-regulation of several pro-inflammatory substrates, such as interleukins (IL-1β, IL-6, IL-8), macrophage inflammatory proteins, ROS, TNF-α and TNF-β, prostaglandins, and so on. Apart from this, ASU unsaponifiable extract also finds its utility in menopausal-associated issues. ASU, with interesting contents of phytoestrogens, tocopherols and tocotrienols contents, has a remarkable positive effect in reducing symptoms related to autoimmune, osteoarticular and menopausal disorders.

## Figures and Tables

**Table 1:** Chemical structure of tocopherols and tocotrienols. Tocopherols have a saturated phytyl tail.

**Form**	**R1**	**R2**	**R3**
α-Tocopherol	CH_3_	CH_3_	CH_3_
β-Tocopherol	CH_3_	CH_3_	H
γ-Tocopherol	H	CH_3_	CH_3_
δ-Tocopherol	H	CH_3_	H
Basic structure of tocotrienols	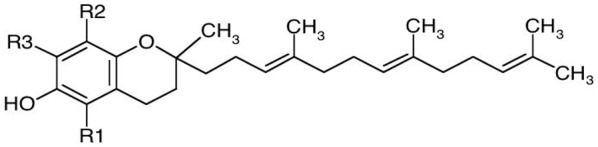		
**Form**	**R1**	**R2**	**R3**
α-Tocotrienol	CH_3_	CH_3_	CH_3_
β-Tocotrienol	CH_3_	H	CH_3_
γ-Tocotrienol	H	CH_3_	CH_3_
δ-Tocotrienol	H	H	CH_3_

**Table 2:** Summary of pharmacological activities of avocado and soybean unsaponifiables (ASU).

Disease	Main Pharmacological Mechanism			Effects	References
**Osteoartrithis**	modifies the structure of the articular cartilage	stimulates the synthesis of articular chondrocytes (anabolic effect)		promotion of cartilage repair reduce stiffness and pain	[5,58,63,64,65,67,68,75,76,77,78,79,80,81]
		Target tissue organ	Molecular mediators		
		Articular synoviocytes, Chondrocytes Chondrocytes, subchondral bone osteoblasts	Collagen II mRNA Aggrecan proteoglycan TGF-β3, Osteocalcin		
		reduces the degradation of articular chondrocytes (anti-catabolic effects)		inhibition cartilage distruction inhibitory effects on inflammatory and catabolic mediators	
		Target tissue organ	Molecular mediators		
		Chondrocytes, Synoviocytes Chondrocytes Fibroblasts, Chondrocytes Chondrocytes, Monocyte/Macrophage-like cells Nuclear translocation of p65 Subchondral bone osteoblasts	IL-Iβ, IL-4, IL-6 IL-8, MIP-1β, MMP13, TNF-α, Fibronectin MMP-2, MMP-3, COX2, PGE2, iNOS, NO Nf-kB TIMP-1		
**Scleroderma**	potential increasing of collagen solubility and reducing cutaneous fibrosis			reducing clinical symptoms	[82]
**Menopause**	estrogen-like properties preferential binding for estrogen receptor-beta (ER-β) than for estrogen receptor-alpha (ER-α).			positive effects in reducing menopausal-related symptoms	[69,83,84,85,86]

**Legend:** TGF-β3 (Transforming Growth Factor-β3), IL-Iβ (interleukin 1beta), IL-4 (interleukin 4), IL-6 (interleukin 6), IL-8 (interleukin 8), MIP-1β (Macrophage inflammatory protein-1 beta), MMP-13 (Matrix metalloproteinase 13), TNFα (Tumor necrosis factor-α), MMP-2 (Matrix metalloproteinase 2), MMP-3 (Matrix metalloproteinase 3), COX-2 (Cyclooxygenase-2), PGE2 (Prostaglandin-E2), iNOS (Inducible nitric oxide synthase), Nitric oxide (NO), NF-κB (nuclear factor kappa B), TIMP-1 (Tissue Inhibitor of metalloproteinases-1).
